# Intrathecal IgG4 synthesis in IgG4 related spinal hypertrophic pachymeningitis: a case report

**DOI:** 10.1186/s42466-024-00343-2

**Published:** 2024-08-29

**Authors:** Lucia K. Feldmann, Regina von Manitius, Birgit Julia Grassmann, Judith Rösler, Julia Onken, Christian Meisel, Arend Koch, Eberhard Siebert, Klemens Ruprecht, Andreas Meisel

**Affiliations:** 1 Department of Neurology with Experimental Neurology, Charité – Universitätsmedizin Berlin, Charitéplatz 1, 10117 Berlin, Germany; 2 Department of Neuropathology, Charité – Universitätsmedizin Berlin, Charitéplatz 1, 10117 Berlin, Germany; 3 Department of Neurosurgery, Charité – Universitätsmedizin Berlin, Charitéplatz 1, 10117 Berlin, Germany; 4 Institute of Medical Immunology, Charité – Universitätsmedizin Berlin, Berlin, Germany; 5 Department of Immunology, Labor Berlin-Charité Vivantes, Berlin, Germany; 6 Department of Neuroradiology, Charité – Universitätsmedizin Berlin, Charitéplatz 1, 10117 Berlin, Germany; 7 Neuroscience Clinical Research Center, Charité – Universitätsmedizin Berlin, Charitéplatz 1, 10117 Berlin, Germany; 8 Center for Stroke Research Berlin, Charité – Universitätsmedizin Berlin, Charitéplatz 1, 10117 Berlin, Germany

**Keywords:** IgG4 related disease, Hypertrophic pachymeningitis, Spinal cord, Plasma cells

## Abstract

Immunoglobulin G4 (IgG4) related hypertrophic pachymeningitis of the spinal cord is a rare condition, characterized by infiltration of the spinal meninges with IgG4-producing plasma cells and subsequent hypertrophic fibrosis. Here, we report on a 65-year-old woman with IgG4 associated hypertrophic spinal pachymeningitis, in whom cerebrospinal fluid (CSF) analysis was a decisive diagnostic tool. Not only could we demonstrate an intrathecal IgG4 production, but also IgG4 positive plasma cells in CSF. Following decompressive surgery, diagnosis of IgG4 associated hypertrophic pachymeningitis was confirmed histologically. Surgery and immunosuppressive therapy with rituximab were associated with clinical improvement. This case highlights CSF analyses as diagnostic tool for detection of IgG4 related hypertrophic pachymeningitis.

Immunoglobulin G4 (IgG4) related disease is an immune mediated fibroinflammatory disease with various clinical manifestations, including hypertrophic pachymeningitis [1]. Rarely, hypertrophic pachymeningitis can occur in the spinal cord, where it is characterized by infiltration of the spinal meninges with IgG4 producing plasma cells and subsequent hypertrophic fibrosis [2]. Consecutive dural thickening may result in spinal cord compression. Diagnosis of IgG4 related hypertrophic pachymeningitis is typically based on biopsies. Here, we report on a patient in whom cerebrospinal fluid (CSF) analysis played a key role in the diagnosis of spinal IgG4 associated hypertrophic pachymeningitis.

A 65-year-old woman with a history of rheumathoid arthritis and hypertension developed pain in the left arm, followed by progressive numbness and gait problems over the course of 4 months. Neurological examination revealed numbness and pallhypesthesia below Th8, proximal leg weakness, brisk reflexes, positive pyramidal signs and a sensory ataxia.

Spinal MRI demonstrated marked dural thickening extending over 13 vertebral segments with consecutive compressive myelopathy. A cranial MRI showed no affection of the cranial meninges or other pathological findings (Fig. 1).

**Fig. 1:**
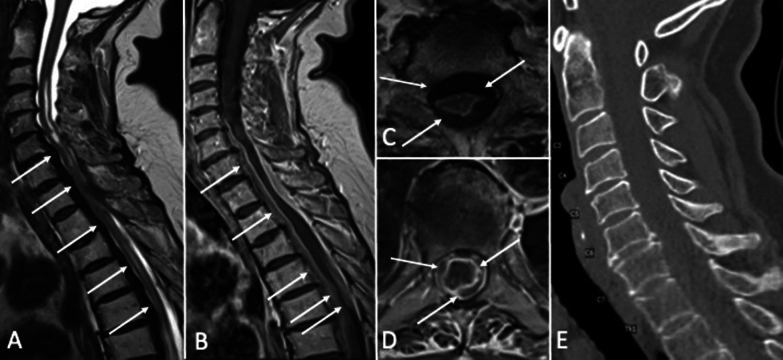
Myelopathy secondary to marked dural thickening. **A**, **C** T2-weighted images showing hypointense circular dural thickening extending over 13 vertebral segments (arrows) causing compressive myelopathy. **B**, **D** Contrast enhanced T1-weighted images showing enhancement of the dural abnormality (arrows). **E** Sagittal CT reconstruction shows no bony abnormality within the spinal canal

CSF examination showed a lymphocytic pleocytosis with 125 cells/µl (reference < 5 cells/ µl), and massively elevated protein levels (32.6 g/l, reference 150–450 mg/l). Remarkably, CSF IgG4 levels were higher in CSF (2.6 g/l) than in serum (1 g/l (reference 0.052–1.25 g/l)), proving intrathecal IgG4 synthesis. Furthermore, immunocytochemistry of plasma cells in CSF showed single plasma cells with strong IgG4 immunoreactivity (Fig. 2A).

**Fig. 2:**
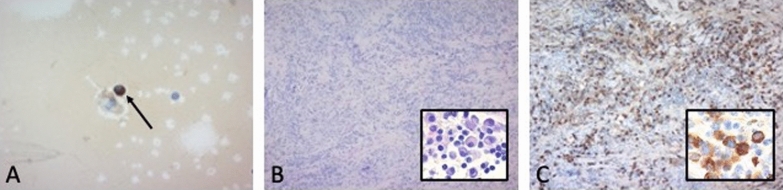
IgG4 producing plasma cells in the cerebrospinal fluid and intradural tissue. **A** Cytologic examination of cerebrospinal fluid (CSF) revealed a marked immunoreactivity of plasma cells stained with an IgG4 antibody. Lymphocytic pleocytosis with IgG4 producing plasma cells (IgG4 immunohistochemistry, 40-fold magnification). **B**, **C** Histologic examination of the surgically removed intradural tissue revealed an inflammatory process characterized by an increased number of IgG4 positive plasma cells ($\approx$ 25% of plasma cells, > 50% IgG4/IgG ratio) (**B** H&E stain, **C** IgG4 immunohistochemistry, tenfold magnification, in the insets 40-fold magnification)

Due to progressive spinal cord compression, surgical decompression with dorsal cervical stabilization was performed. Immunohistochemistry revealed an inflammatory, plasma cell dominated process, with ≈ 25% IgG4 positive cells (Fig. 2B, C), confirming the diagnosis of IgG4 related spinal hypertrophic pachymeningitis [3].

Immunotherapy with i.v. rituximab (1000 mg) was initiated at six-monthly intervals. At one-year-follow-up, both a clinical (reduced pallhypesthesia, slight improvement of gait disorder) and neuroradiological (reduced contrast uptake of the spinal meninges) improvement could be observed.

This case highlights the usefulness of CSF analysis in the diagnosis of IgG4 related hypertrophic pachymeningitis. Previously, IgG4 CSF/plasma ratios have been discussed as potential biomarkers for IgG4 related pachymeningitis [4]. Nevertheless, CSF IgG4 assessments have only rarely been reported so far. A case collection showed increased intrathecal IgG4 ratios in all 3 patients [3]. A recent review analyzing data from 60 patients reported CSF IgG4 levels in only four patients [5]. In these four patients CSF IgG4 levels were on average more than tenfold higher than the upper limit of normal (cut off 3.2 mg/l). This indicates that CSF analysis is a valuable diagnostic tool, which has already been described as having high sensitivity and specificity [4], but is still used too rarely.

The patient reported herein had a remarkably elevated CSF protein, very likely due to the meningeal hypertrophy with resulting compressive myelopathy and a consecutively trapped lumbar CSF. Nevertheless, it is highly unlikely that the key finding of our study, i.e. a higher level of IgG4 in CSF than in serum is explained by a trapped lumbar CSF. Indeed, immunoglobulins present in the CSF normally reach the CSF by passive diffusion from blood. Thus, even in situations with a very pronounced blood–brain barrier impairment, such as a trapped lumbar CSF, immunoglobulin levels in CSF are not expected to be higher than those in serum. Therefore, the higher IgG4 levels in CSF than in serum observed in our patient strongly suggest an intrathecal production of IgG4 by IgG4 positive plasma cells, which migrated into the CSF.

An important addition to the pathophysiology and diagnosis of this disease is that we were also able to detect IgG4 positive plasma cells in CSF, as a further diagnostic confirmation that requires only the CSF as a comparatively non-invasive examination.

As in our case, treatment generally includes surgical decompression and immunotherapy. Out of 6 reported patients receiving rituximab, all showed clinical improvement without recurrence [6]. Interestingly, a recent case reported symptom improvement upon intrathecal rituximab therapy in a patient with slowly progressive intracranial affection, who had been treatment resistant to intravenous rituximab therapy [7].

Overall, this case underscores the potential value of CSF diagnostics for the diagnosis of IgG4 related hypertrophic pachymeningitis, a rare, but treatable neurological condition.

## Data Availability

Data sharing is not applicable to this article as no datasets were generated or analyzed during the current study.
